# Airway allergy causes alveolar macrophage death, profound alveolar disorganization and surfactant dysfunction

**DOI:** 10.3389/fimmu.2023.1125984

**Published:** 2023-05-10

**Authors:** Lidia Feo-Lucas, Cristina Godio, María Minguito de la Escalera, Natalia Alvarez-Ladrón, Laura H. Villarrubia, Adrián Vega-Pérez, Leticia González-Cintado, Jorge Domínguez-Andrés, Belén García-Fojeda, Carlos Montero-Fernández, Cristina Casals, Chiara Autilio, Jesús Pérez-Gil, Georgiana Crainiciuc, Andrés Hidalgo, María López-Bravo, Carlos Ardavín

**Affiliations:** ^1^ Departamento de Inmunología y Oncología, Centro Nacional de Biotecnología/ Consejo Superior de Investigaciones Científicas (CSIC), Madrid, Spain; ^2^ Departamento de Bioquímica y Biología Molecular, Facultad de Biología, Universidad Complutense, Madrid, Spain; ^3^ Instituto de Investigación Sanitaria Hospital 12 de Octubre i+12, Madrid, Spain; ^4^ Centro Nacional de Investigaciones Cardiovaculares Carlos III, Madrid, Spain

**Keywords:** alveolar macrophages (AM), monocytes, airway allergy inflammation, allergic asthma, alveolar dysfunction, pneumocyte hypertrophy, surfactant dysfunction

## Abstract

Respiratory disorders caused by allergy have been associated to bronchiolar inflammation leading to life-threatening airway narrowing. However, whether airway allergy causes alveolar dysfunction contributing to the pathology of allergic asthma remains unaddressed. To explore whether airway allergy causes alveolar dysfunction that might contribute to the pathology of allergic asthma, alveolar structural and functional alterations were analyzed during house dust mite (HDM)-induced airway allergy in mice, by flow cytometry, light and electron microscopy, monocyte transfer experiments, assessment of intra-alveolarly-located cells, analysis of alveolar macrophage regeneration in *Cx3cr1* ^cre^:*R26-yfp* chimeras, analysis of surfactant-associated proteins, and study of lung surfactant biophysical properties by captive bubble surfactometry. Our results demonstrate that HDM-induced airway allergic reactions caused severe alveolar dysfunction, leading to alveolar macrophage death, pneumocyte hypertrophy and surfactant dysfunction. SP-B/C proteins were reduced in allergic lung surfactant, that displayed a reduced efficiency to form surface-active films, increasing the risk of atelectasis. Original alveolar macrophages were replaced by monocyte-derived alveolar macrophages, that persisted at least two months after the resolution of allergy. Monocyte to alveolar macrophage transition occurred through an intermediate stage of pre-alveolar macrophage and was paralleled with translocation into the alveolar space, Siglec-F upregulation, and downregulation of CX3CR1. These data support that the severe respiratory disorders caused by asthmatic reactions not only result from bronchiolar inflammation, but additionally from alveolar dysfunction compromising an efficient gas exchange.

## Introduction

Airway allergy is triggered after contact of allergens with the airway epithelium, inducing complex deleterious innate and adaptive immune responses, that drive pathological changes of lung physiology, that can lead to asthma and eventually to death by anaphylaxis (1). Asthmatic reactions can cause life-threatening respiratory disorders, that have been associated with a severe airway inflammation, involving mucus hypersecretion, subepithelial fibrosis, hyperplasia of smooth muscle and bronchoconstriction, leading to critical narrowing of airway lumen (1). Nevertheless, whether airway allergy cause alveolar dysfunction, and thus compromise gas exchange, remains unaddressed.

Alveoli are essentially composed of alveolar type 1 (AT1) and type 2 (AT2) epithelial cells, responsible for gas exchange and surfactant production and recycling, respectively, alveolar macrophages (AMØs), and capillaries (2). AT1 cells, representing around 10% of alveolar cells and covering more than 95% of the alveolar surface, are essential for gas exchange, whereas AT2 cells, representing around 60% of alveolar cells, but covering less than 5% of the alveolar surface, are responsible for surfactant production and recycling, and for alveolar repair (2). Pulmonary surfactant is composed of complex macromolecular aggregates of lipids and surfactant-associated proteins and has a crucial role in lung physiology by reducing the surface tension at the air-liquid interface existing between alveolar gas and the aqueous hypophase lining the alveolar epithelial cell surface, preventing alveolar and terminal airway collapse at end-expiration (3). AMØs are crucial for defense against lung infections and fulfill a critical function in surfactant recycling, since defective AMØ development or function leads to proteinosis, due to intra-alveolar surfactant accumulation, and consequently to a severe reduction in gas exchange (4). AMØs present in the adult mouse are generated during the embryonic life from yolk sac macrophages and fetal liver monocytes and maintained by self-renewal (5), although it has been recently reported that they become progressively, yet partially, replaced by monocyte-derived AMØs in non-pathological conditions (6).

As pointed out above, whether allergy asthma involve a dysfunction of the alveolar system remains unexplored. Using a mouse model of acute allergic asthma induced by house dust mite (HDM) extracts, our results demonstrate that HDM airway allergy caused a severe alveolar disorganization, involving pneumocyte hypertrophy and thickening of the alveolar lining, associated with profound alterations in the biophysical properties of pulmonary surfactant. Moreover, HDM allergy resulted in a massive elimination of the original embryonic AMØ population which was replaced by a new Ly6C^high^ monocyte-derived AMØ population, that persisted at least two months after the resolution of the allergic reaction, and enabled the recovery of surfactant function.

In conclusion, our data provide, to our knowledge, the first experimental evidence that airway allergic reactions cause alveolar dysfunction and death of alveolar macrophages, and support that the severe respiratory disorders caused by allergic asthma not only result from airway inflammation, but also from profound, previously unknown, alterations in the alveolar system, suggesting that new therapeutic strategies against asthma should be designed based on a combined treatment of bronchiolar inflammation and alveolar dysfunction.

## Materials and methods

### Mice

C57BL/6 CD45.2^+^ (B6 or B6-CD45.2^+^) mice were purchased from Charles River (L’Arbresle, France) and C57BL/6 CD45.1^+^ (B6-CD45.1^+^) mice from Jackson (Maine). LysM-eGFP (7) and CX3CR1-eGFP (8) mice were supplied by A. Hidalgo (CNIC, Spain). 8-10-week old B6, B6-CD45.1^+^ and LysM-eGFP mice were housed at the CNB Animal Facility on a 12/12 light/dark cycle. Littermates of the same sex were randomly assigned to experimental groups. Bone marrow (BM) from *Cx3cr1* ^cre^:*R26-yfp* mice (9) was provided by S. Jung (The Weizmann Institute of Science, Israel). All the experiments were approved by CNB Animal Care Committee (protocol 312/14).

### Induction of HDM-allergy

HDM-allergy was induced following a protocol modified from a protocol described in a previous report from our group (10). In brief, mice received an intraperitoneal injection of 5 x 10^4^ monocyte-derived dendritic cells at day -7, followed, at day 0, by intratracheal administration of 20 μg HDM-extract (*Dermatophagoides pteronyssinus* extract; Greer Laboratories, Lenoir, North Carolina), in a total volume of 40 μl of PBS, under ketamine/xylazine anesthesia. Monocyte-derived dendritic cells were prepared as described (10) and incubated prior to injection for 4 hr with 30 μg/ml HDM extract.

### Cell suspensions

Bronchoalveolar lavage (BAL) was performed with 3 x 1 mL EDTA-containing PBS. BAL cell suspensions were obtained after centrifugation for 5 min at 400g. Lung cell suspensions were obtained after performing a BAL; for this purpose lungs were cut into small pieces, digested with 180 μg/ml Liberase TM and 40 mg/ml DNAse (both from Roche, Mannheim, Germany) for 40 min at 37°C, resuspended in RPMI supplemented with 10% FCS, filtered through 40-μm cell strainers (BD Pharmingen, San Diego, CA) and washed twice in EDTA-containing PBS after erythrocyte lysis by osmotic shock.

### Thorax-shielded BM chimeras

CD45.1/CD45.2 thorax-shielded BM chimeras were established by intravenous (i.v.) injection of 5 x 10^6^ BM cells from C57BL/6 (B6)-CD45.1^+^ mice into 8-week old lethally irradiated B6-CD45.2^+^ mice (single dose 10 Gy γ-radiation, using a JL Shepherd Mark I-30 ^137^Cs irradiator), in which the thorax was lead shield-protected. *Cx3cr1* ^cre^:*R26-yfp*/CD45.1 thorax-shielded BM chimeras were established by i.v. injection of 5 x 10^6^ BM cells from CD45.2^+^ *Cx3cr1* ^cre^:*R26-yfp* mice into 8-week old lethally irradiated, thorax-protected, B6-CD45.1^+^ mice.

### Monocyte transfer

4 x 10^6^ BM Ly6C^high^ monocytes, isolated as described (11), from LysM-eGFP mice, at d1 of HDM allergy, were transferred intravenously into B6 mice at d1 of HDM allergy.

### Postmortem intratraqueal alveolar cell staining

Mice euthanized by controlled CO_2_ inhalation overdose received 125 ng of Pacific Blue-conjugated anti-CD45 intratracheally (i.t.) in a total volume of 1 ml (corresponding to a 1/4.000 dilution); control mice received 1 ml of PBS intratracheally. The concentration and volume of Pacific Blue-conjugated anti-CD45 was determined in titration experiments in order to ensure an efficient staining of AMØs while avoiding the diffusion of the antibody into the lung parenchyma. After 1 min intra-alveolarly-located cells were analyzed as described below.

### Flow cytometry

Analysis of lung and BAL cell suspensions was performed after seven-color staining with FITC-conjugated anti-MHCII (clone 2G9; BD Pharmingen), PECy7-conjugated anti-CD11b (clone M1/70; eBioscience, San Diego, CA) or PECy7-conjugated anti-Ly-6G (clone 1A8; BD Pharmingen), APC-conjugated anti-CD64 (clone X54-5/7.1; Biolegend, San Diego, CA), APC-Cy7-conjugated anti-CD11c (clone HL3; BD Pharmingen), PE-conjugated anti-Siglec-F (E50-2440; BD Pharmingen), Pacific Blue-conjugated anti-CD45 (clone 30-F11; Biolegend) and biotin-conjugated anti-Ly6C (clone Al-21) followed by streptavidin-PerCP (BD Pharmingen). Analysis of dead AMØs was performed after seven-color staining with PECy7-conjugated anti-Ly-6G, PE-conjugated anti-Siglec-F, Pacific Blue-conjugated anti-CD45, PerCP/Cy5.5-conjugated anti-CD64 (clone X54-5/7.1; Biolegend) and biotin-conjugated anti-Ly6C, followed by streptavidin-APC-Cy7 (Biolegend). Cells were then resuspended at 1 × 10^6^ cells/ml in 100 μl binding buffer (Biolegend) and incubated with FITC-conjugated Annexin-V (Biolegend) for 15 min at 4 °C in the dark. AMØs death was assessed by Annexin-V staining after gating on AMØs. Analysis of CD45.1/CD45.2 BM chimeras was performed after seven-color staining with FITC-conjugated anti-MHCII, PECy7-conjugated anti-Ly-6G, APC-conjugated anti-CD64, PE-conjugated anti-Siglec-F, Brilliant Violet 421-conjugated anti-CD45.1 (clone A20; Biolegend), PerCP/Cy5.5-conjugated anti-CD45.2 (clone 104; Biolegend) and biotin-conjugated anti-Ly6C, followed by streptavidin-APC-Cy7. Analysis of *Cx3cr1* ^cre^:*R26-yfp*/CD45.1 BM chimeras was performed after seven-color staining with PECy7-conjugated anti-CD11b, APC-conjugated anti-CD64, PE-conjugated anti-Siglec-F and anti-Ly6G, Brilliant Violet 421-conjugated anti-CD45.1, PerCP/Cy5.5-conjugated anti-CD45.2 and biotin-conjugated anti-Ly6C, followed by streptavidin-APC-Cy7, or after seven-color staining with PECy7-conjugated anti-CD11b, APC-conjugated anti-CD64, PE-conjugated anti-Siglec-F, Brilliant Violet 421-conjugated anti-CD45.1, PerCP/Cy5.5-conjugated anti-CD45.2 and biotin-conjugated anti-MHC-II, followed by streptavidin-APC-Cy7. Analysis of cells derived from intravenously-transferred LysM-eGFP-monocytes was performed after six-color staining with PECy7-conjugated anti-Ly-6G, APC-conjugated anti-MHC-II (clone M5/114.15.2; eBioscience), PE-conjugated anti-Siglec-F, Pacific Blue-conjugated anti-CD45, PerCP/Cy5.5-conjugated anti-CD64 and biotin-conjugated anti-Ly6C, followed by streptavidin-APC-Cy7. Analysis of intra-alveolarly-located cells was performed after seven-color staining with FITC-conjugated anti-MHCII, PECy7-conjugated anti-CD11b, APC-conjugated anti-CD64, APC-Cy7-conjugated anti-CD11c, PE-conjugated anti-Siglec-F, PerCP/Cy5.5-conjugated anti-CD45.2 and biotin-conjugated anti-Ly6C, followed by streptavidin-APC-Cy7.

Autofluorescence was detected using a 525/50-nm band pass filter after excitation with a 405 nm-violet laser.

Analysis of CD206 expression was performed after seven-color staining with FITC-conjugated anti-MHCII, PECy7-conjugated anti-Ly-6G, PE-conjugated anti-Siglec-F, Pacific Blue-conjugated anti-CD45, PerCP/Cy5.5-conjugated anti-CD64 and biotin-conjugated anti-Ly6C, followed by streptavidin-APC-Cy7. Subsequently, cells were fixed in Cytofix/Cytoperm solution (BD Pharmingen) and incubated with APC-conjugated anti-CD206 (clone C068C2; Biolegend). Data were acquired on a LSRII cytometer (BD Biosciences, San José, CA) and analyzed using FlowJo X software (Tree Star, Ashland, OR).

### Electron microscopy

Lungs were fixed with 2.5% glutaraldehyde and 4% paraformaldehyde in PBS for 2 hours at RT and overnight at 4°C, postfixed with 1% osmium tetroxide/0,8% potassium ferricyanide for 1 h at 4°C, dehydrated in graded acetone solutions and embedded in Epon-812 (TAAB Laboratories Ltd., Berkshire, UK). Semi-thin sections (1 µm) were obtained with a Leica EM UC6 ultramicrotome and stained with toluidine blue. Images were acquired with a Leica DM2500 microscope (Leica, Wetzlar, Germany). Ultra-thin sections (80 nm) were obtained with a Leica EM UC6 ultramicrotome, counterstained with uranyl acetate and lead citrate and examined with a Jeol 1011 transmission electron microscope (Tokyo, Japan).

### Biochemical analysis of BAL

Cell-free BAL supernatant was obtained by centrifugation for 10 min at 700 g at 4°C. Total BAL protein concentration was measured in cell-free BAL supernatants by Lowry’s method modified by adding sodium dodecyl sulphate (SDS). Lipid extraction was performed by chloroform and methanol extraction and quantitation of BAL phospholipids was achieved by phosphorus analysis, as described (12).

### Analysis of surfactant proteins by western blot

Electrophoretic analysis of BAL proteins was performed under reducing conditions (5% β-mercaptoethanol) by one-dimensional SDS/PAGE, using running gels of 12% for SP-A and SP-D, 16% for SP-B and 18% for SP-C. In the case of SP-A and SP-D, the same amount of BAL protein was applied for all samples. In case of SP-B and SP-C, the same amount of BAL phospholipids was applied for all samples. After electrophoresis, samples were transferred to polyvinylidene fluoride membranes (Bio-Rad Laboratories, Hercules, CA). Blotting was performed as previously described (12) SP-A and SP-B, were detected using anti-human-SP-A and anti-porcine SP-B polyclonal antibodies prepared in our laboratory. SP-C was detected using and an anti-recombinant human SP-C polyclonal antibody kindly provided by Nycomed Pharma (Konstanz, Germany). Secondary HRP-conjugated anti-rabbit IgG antibodies were from Cell Signaling Technologies, (Danvers, MA). SP-D was detected with an anti SP-D mAb (clone 1A10A9, Seven Hills Bioreagents, Cincinnati, OH), followed by a secondary anti-mouse IgG antibody (Sigma-Aldrich, St. Louis, MO). Proteins were visualized using chemiluminiscence detection (Amersham Hyperfilm ECL, GE Healthcare, Little Chalfont, UK). Protein bands were quantified by densitometry using the Quantity One software (Bio-Rad Laboratories).

### Analysis of surfactant biophysical properties

Total surfactant complexes (large + small aggregates) were precipitated from cell-free BAL supernatants by ultracentrifugation for 60 min at 100,000 g at 4°C, and diluted to 10 mg/mL phosphatidylcholine concentration with Tris buffer (5mM; pH7) containing 150 mM NaCl (Sigma-Aldrich). Phosphatidylcholine concentration was measured using enzymatic methods (Spinreact, Gerona, Spain) as previously published (13). The interfacial activity of surfactant samples was assessed by captive bubble surfactometry, a technique allowing to analyze surfactant function during breathing-like compression-expansion cycles (14). To this end, 200 nL of surfactant, at 10 mg/mL, was applied at the surface of an air bubble in a Captive Bubble Surfactometer (CBS) at a frequency of 30 compression-expansion cycles/min, as described in **Supplementary Figure 1**. Measurements were performed in triplicate for each surfactant sample.

### Quantification and statistical analysis

Data are presented as mean ± SD (or mean ± SEM), as indicated in the legend of each figure. Statistical parameters including the exact value of n, precision measures (mean ± SD or SEM) and statistical significance are reported in the Figures and the Figure Legends when necessary. Data are judged to be statistically significant when p < 0.05 by two-tailed Student’s t test. In figures, asterisks denote statistical significance (*, p < 0.05; **, p < 0.01; ***, p < 0.001). Statistical analysis was performed using Microsoft Excel or GraphPad PRISM 6 softwares.

## Results

### HDM allergy caused alveolar disorganization and AMØ disappearance

**Figure 1:**
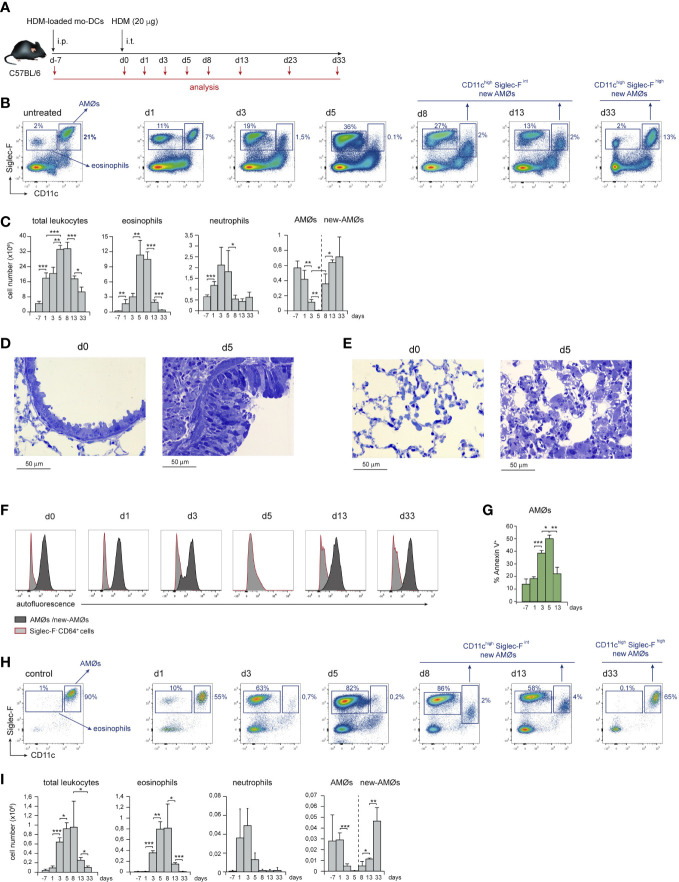
HDM allergy caused alveolar disorganization and AMØ disappearance. **(A)** Protocol of induction of HDM allergy used in this study; i.p.: intraperitoneal; i.t.: intratracheal; mo-DCs: monocyte-derived dendritic cells **(B)** Flow cytometry (FACS) analysis of AMØs and eosinophils in lung cell suspensions at the indicated times. **(C)** Absolute number per mouse of the indicated lung cell populations. **(D)** Lung semi-thin sections showing the increase in airway mucus production and peribronchiolar fibrosis from d0 to d5. Toluidine blue staining. **(E)** Lung semi-thin lung sections showing the alveolar disorganization and pneumocyte hypertrophy from d0 to d5; toluidine blue staining. **(F)** FACS analysis of autofluorescence of AMØs and Siglec-F^-^ CD64^+^ cells (monocytes and moCs). **(G)** Quantification of Annexin-V^+^ AMØs. **(H)** FACS analysis of AMØs and eosinophils in BAL cell suspensions at the indicated times. **(I)** Absolute cell number per mouse of the indicated BAL cell populations. In C and I the percentage of each population among all CD45^+^ lung cells is indicated. Similar results were obtained in 5 independent experiments.

Airway allergy was induced by HDM following the protocol described in **Figure 1A**. HDM-driven allergy caused an airway inflammation similar to human allergic asthma (15), that involved a massive leukocyte infiltration into the lung and eosinophilia, peaking at day 5 (d5) after i.t. HDM-extract challenge (**Figures 1B**, **C**), paralleled by bronchiolar inflammation, characterized by increased mucus production and fibrosis (**Figure 1D**). Unexpectedly, HDM allergy caused a profound disorganization of the alveolar system leading to reduction of the alveolar space (**Figure 1E**). Neutrophils were also actively recruited by d1, but their number dropped by d8 (**Figure 1C**). Analysis of lung cell suspensions revealed that the AMØ population, characterized as CD11c^high^ Siglec-F^high^ autofluorescent cells (**Figures 1B, F**), was markedly reduced at d3, and almost undetectable at d5 (**Figures 1B, C**). AMØ reduction was concomitant with an increase in eosinophils (**Figure 1C**) and Annexin-V^+^ AMØs (**Figure 1G**), suggesting the reduction in AMØs was due to cell death. AMØ disappearance was also noticeable in the BAL that contained a large number of eosinophils at d5-d8 (**Figures 1H, I**). Importantly, only a fraction of AMØs can be harvested by BAL (〜10-15% of total AMØs), either in control or allergic mice, indicating that AMØ studies have to be performed in lung cell suspensions, but not in BAL cell suspensions.

From d8 to d13, eosinophils dropped significantly, and a population of autofluorescent CD11c^high^ cells with lower Siglec-F expression than control AMØs, although comparable in number, was detectable in lung (**Figures 1B, C**) and BAL cell suspensions (**Figures 1H, I**) indicating that, at least in part, CD11c^high^ Siglec-F^int^ cells were intra-alveolarly located. By d33, a population of AMØs expressing CD11c and Siglec-F levels matching those of control AMØs was noticeable, and AMØ and eosinophil number returned to basal levels (**Figures 1B, C**). These data led us to hypothesize that at the peak of eosinophilia and alveolar disorganization, HDM allergy caused the death of original AMØs, that were replaced by new-AMØs.

### AMØs newly-formed during the resolution of HDM allergy derived from monocytes

**Figure 2:**
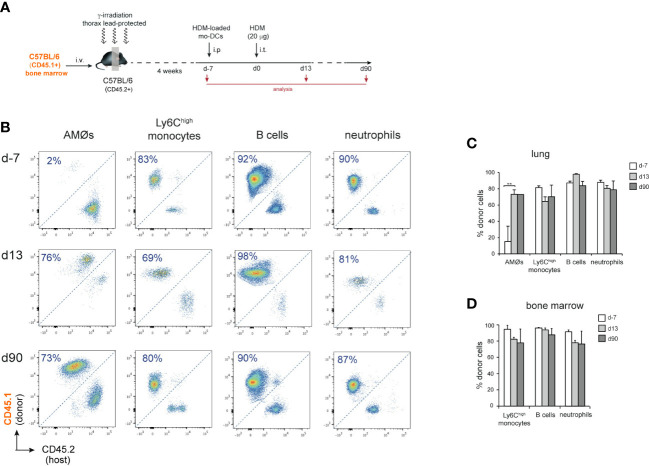
AMØs were newly-formed from BM precursors during HDM allergy. **(A)** Protocol for the induction of HDM allergy in CD45.1/CD45.2 thorax-shielded BM chimeras. **(B)** FACS analysis of CD45.1/CD45.2 thorax-shielded BM chimeras at the indicated times. **(C)** Quantification of donor-host chimerism of the indicated lung cell populations of CD45.1/CD45.2 thorax-shielded BM chimeras at the indicated times. **(D)** Quantification of donor-host chimerism of the indicated BM cell populations at the indicated times; data are expressed as mean ± SD of 4 mice per condition. Similar results were obtained 2 independent experiments.

To address whether new-AMØs were originated by self-renewal from original embryonic-derived AMØs, or *de novo* from BM progenitors, AMØ regeneration was analyzed CD45.1/CD45.2 chimeric mice, in which the hematopoietic system was mainly of donor origin, while AMØs were host-derived. To this end, BM cells from B6-CD45.1^+^ mice were transferred into B6-CD45.2^+^ mice, that were subjected to lethal γ-irradiation after protecting the thorax with a lead shielding. (**Figure 2A**). At d-7, 〜90% AMØs were of host-derived, whereas 〜80-90% of lung Ly6C^high^ monocytes (hereafter monocytes), B cells and neutrophils, were donor-derived (**Figures 2B, C**). A comparable chimerism was found in BM monocytes, B cells and neutrophils during HDM allergy (**Figure 2D**), reflecting that the lead shield had provided protection not only to AMØs, but also to thoracic hematopoietic foci that generated 〜10-20% host-derived monocytes, B cells and neutrophils. At d13〜70% AMØs were donor-derived (corresponding to 〜90% BM origin, normalized BM monocyte chimerism), revealing that original AMØs were almost entirely replaced during the allergic reaction by a new, BM-derived, AMØ population (**Figures 2B, C**). Interestingly, 2 months after allergy resolution 〜70% of AMØs were still of donor origin (corresponding to 〜95% BM origin, normalized BM monocyte chimerism), revealing that BM-derived AMØs persisted long after allergy had subsided (**Figures 2B, C**).

Under experimental or pathological conditions new tissue-resident macrophages, such as Kupffer cells, microglial cells, large peritoneal macrophages or AMØs can be generated from monocytes (16).

**Figure 3:**
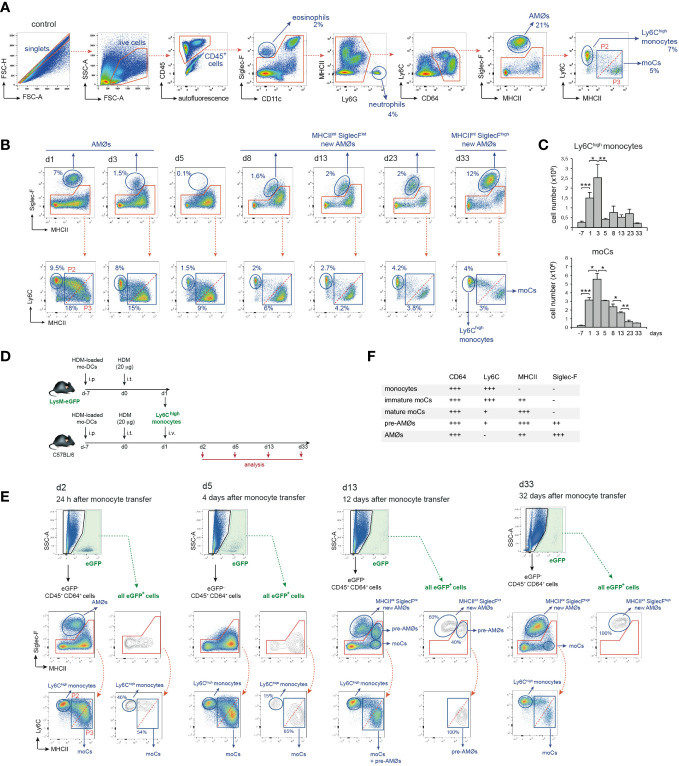
Intravenously transferred monocytes generated AMØs during HDM allergy. **(A)** FACS gating strategy used to analyze lung cell suspensions; the percentage of each population among all CD45^+^ lung cells is indicated. **(B)** Flow cytometry analysis of AMØs, Ly6C^high^ monocytes and mo-Cs at the indicated times, performed as indicated in A; the percentage of monocytes and moCs among all CD45^+^ lung cells is indicated. **(C)** Absolute number per mouse of Ly6C^high^ monocytes and mo-Cs at the indicated times. **(D)** Protocol of intravenous transfer of monocytes from LysM-eGFP mice into B6 mice at d1 of HDM allergy; i.p.: intraperitoneal; i.t.: intratracheal; i.v.: intravenous. **(E)** FACS analysis of the progeny of intravenously transferred eGFP^+^ monocytes at the indicated times; the percentage of eGFP^+^ populations is indicated. **(F)** Phenotype of monocytes, moCs, pre-AMØs and AMØs. In **(A, B, E)**, diagonal dashed red lines, in MHCII vs Ly6C plots, define immature moCs (P2; upper left) and mature moCs (P3; lower right). Similar results were obtained 5 independent experiments **(A–C)** and in 2 independent experiments **(D, E)**.

To assess whether AMØs newly-formed during HDM allergy were derived from monocytes, we first analyzed the kinetics of monocytes and monocyte-derived cells (moCs), defined following the gating strategy described in **Figure 3A**. In steady state, lung mature moCs were characterized as CD64^+^ Ly6C^low^ MHC class-II (MHCII)^high^ Siglec-F^-^ cells, as reported (17). Monocytes (defined as CD64^+^ Ly6C^high^ MHCII^-^ Siglec-F^-^ cells) were actively recruited to the lung at d1-d3, and dropped by d5 (**Figures 3B, C**). Monocyte differentiation into moCs, occurred through an intermediate stage of CD64^+^ Ly6C^high^ MHCII^low^ Siglec-F^-^ immature moCs (P2; **Figure 3B**). moCs peaked at d3 and diminished progressively as allergy declined (**Figures 3B, C**), suggesting that moCs were generated after a single wave of monocyte recruitment.

To address whether monocytes recruited to the lung were able to generate AMØs during HDM allergy, fluorescence-trackable BM monocytes were isolated from LysM-eGFP mice at d1 after HDM challenge, i.e. when monocytes started to be recruited (**Figure 3C**), and transferred i.v. into B6 mice at d1 (**Figure 3D**). At d2, 24 h after transfer, 〜50% eGFP^+^ cells were monocytes, and 〜50% were Ly6C^high^ MHCII^int^ Siglec-F^-^ immature moCs (P2; **Figure 3E**). At d5, 〜15% eGFP^+^ cells were monocytes and 〜85% were Ly6C^low^ MHCII^high^ Siglec-F^-^ mature moCs. At d13 〜60% of lung eGFP^+^ cells were MHC II^int^ Siglec-F^int^ new-AMØs, whereas the rest were Ly6C^low^ MHCII^high^ moCs expressing intermediate levels of Siglec-F, that based on their phenotype, kinetics and alveolar location (as demonstrated later in this report) were immature AMØs, hereafter pre-AMØs. At this stage, endogenous moCs could be subdivided in Ly6C^low^ MHCII^high^ Siglec-F^int^ pre-AMØs and Ly6C^low^ MHCII^high^ Siglec-F^-^ mature moCs (**Figure 3E**). By d33, all lung eGFP^+^ cells were fully differentiated MHCII^int^ Siglec-F^high^ new-AMØs, pre-AMØs being no longer detectable (**Figure 3E**), supporting that new-AMØs were generated from monocytes, through an intermediate Ly6C^low^ MHCII^high^ Siglec-F^int^ pre-AMØ population. The phenotype of monocytes, moCs, pre-AMØs and AMØs is summarized in **Figure 3F**.

**Figure 4:**
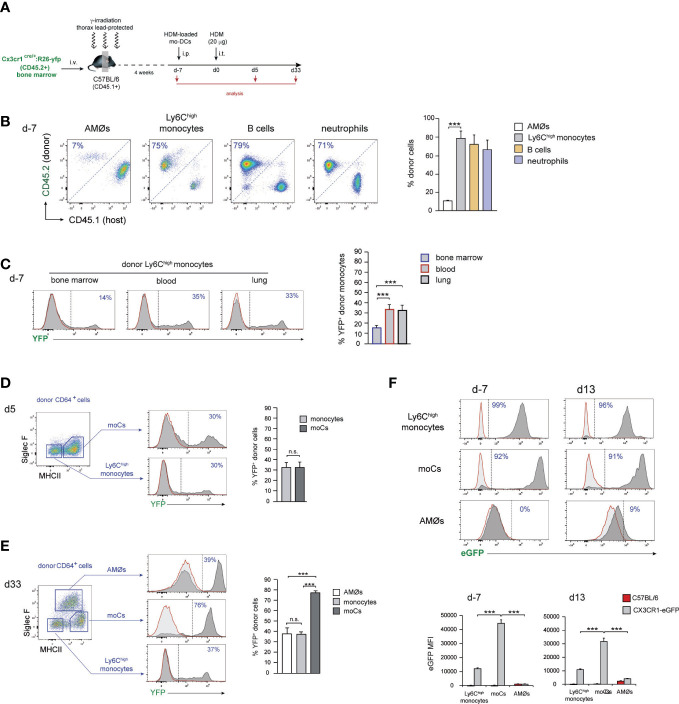
AMØs newly-formed during the resolution of HDM allergy derived from monocytes. **(A)** Protocol for the induction of HDM allergy in *Cx3cr1* ^cre^:*R26-yfp*/CD45.1 thorax-shielded BM chimeras. **(B)** Quantification of donor-host chimerism of the indicated lung cell populations at d-7 of HDM allergy. **(C)** Quantification of YFP expression by BM, blood and lung donor-type monocytes at d-7 of HDM allergy. **(D)** FACS analysis and quantification of YFP expression by the indicated lung cell populations at d5 of HDM allergy. **(E)** FACS analysis and quantification of YFP expression by the indicated lung cell populations at d33 of HDM allergy. **(F)** FACS analysis and quantification of CX3CR1 expression by monocytes, moCs and AMØs, at the indicated days during HDM allergy in CX3CR1-eGFP mice, assessed by the expression of eGFP. Quantification of CX3CR1 expression was based on the mean fluorescence intensity (MFI) of eGFP expression. In **(D–F)**, dark grey profiles correspond to YFP (or eGFP in **F**) expression, and red lined light grey profiles correspond to background staining in B6 mice; the percentage of YFP^+^ cells (or eGFP^+^ cells in **F**) is indicated. Data are expressed as mean ± SD of 6 mice per condition.

Monocyte transfer experiments allowed to assess the ability of exogenous monocytes to be recruited to the lung and to monitor the different phases of their differentiation into AMØs, but do not provide a definitive demonstration of the monocytic origin of endogenous new-AMØs generated during HDM allergy. This issue was addressed analyzing AMØ regeneration in *Cx3cr1* ^cre^:*R26-yfp*/CD45.1 BM chimeras (**Figure 4A**), in which CX3CR1 promoter-driven Cre recombinase expression leads to the rearrangement of the YFP reporter locus, establishing a stable fluorescent labeling (9). As expected, 4 weeks after BM transfer, 〜90% AMØs were of host origin whereas 〜70-80% monocytes, B cells and neutrophils, were donor-derived (**Figure 4B**). At this time point, 〜15% donor-derived BM monocytes and 〜30% donor-derived blood and lung monocytes were YFP^+^ (**Figure 4C**), most likely due to their short life span not allowing for 100% rearrangement of YFP locus. At d5, 〜30% donor-derived lung monocytes expressed YFP, and a comparable YFP expression was found on donor-derived moCs (**Figure 4D**), whereas at d33 up to 80% donor-derived moCs expressed YFP (**Figure 4E**), reflecting that moCs generated during the resolution of allergy had a longer life span and maintained the activity of the CX3CR1 promoter, leading to an efficient YFP locus rearrangement. Interestingly, at this time point 〜40% donor-derived lung monocytes and new-AMØs expressed YFP. These results indicate that transition from monocytes to new-AMØs was concomitant with the inactivation of the CX3CR1 promoter and therefore with the shut off of Cre-mediated YFP locus rearrangement. In support of this postulate, CX3CR1 expression was downregulated from monocytes to AMØs, as assessed by the analysis of CX3CR1-eGFP mice during HDM allergy (**Figure 4F**).

### Differentiation of monocytes into pre-AMØs was paralleled by their translocation into the alveolar space

**Figure 5:**
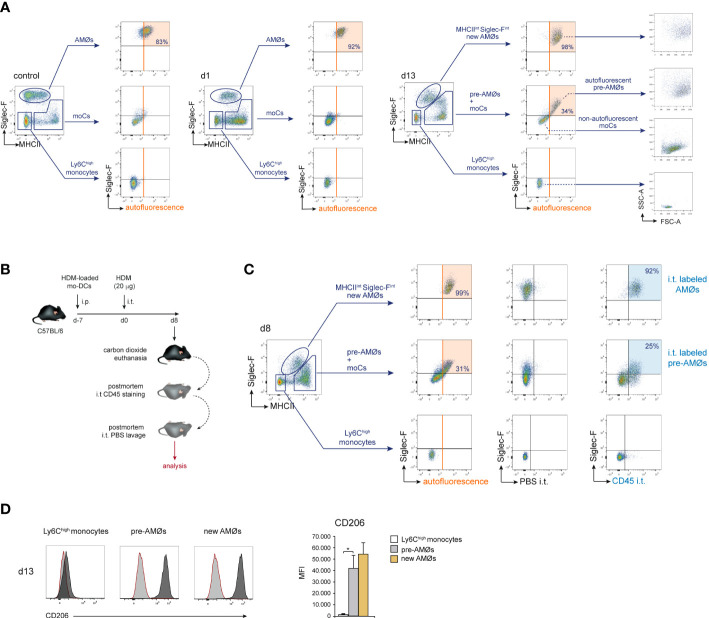
Differentiation of monocytes into AMØs was paralleled by their translocation into the alveolar space. **(A)** FACS analysis of autofluorescence of the indicated lung cell populations, in untreated mice, and at d1 and d13 of HDM allergy; the percentage of autofluorescent cells is indicated. **(B)** Protocol for the staining of intra-alveolarly-located cells by i.t. administration of Pacific Blue-conjugated anti-CD45 antibodies. **(C)** Detection by FACS of lung populations labeled by i.t. administration of Pacific Blue-conjugated anti-CD45 at d8 of HDM allergy; the percentage of cells labeled by intratracheally-administered anti-CD45 is indicated; i.p.: intraperitoneal; i.t.: intratracheal. **(D)** FACS analysis and quantification of CD206 expression by the indicated cell populations at d13 of HDM allergy; dark grey profiles correspond to CD206 expression; red lined light grey profiles correspond to background staining with an isotype control antibody. Data are expressed as mean ± SD of 5 mice per condition.

We next sought to addressed whether monocyte differentiation into AMØs occurred extra- or intra-alveolarly, by assessing the location of pre-AMØs based on their autofluorescence, a characteristic of AMØs associated to their intra-alveolar location, which has been correlated to their exposure to environmental particulate matter (18). In control mice, and at d1 and d13 of HDM allergy, AMØs, but neither monocytes nor MHCII^high^ Siglec-F^-^ moCs, were autofluorescent (**Figure 5A**). However, at d13, both pre-AMØ pre-AMØs and new-AMØs displayed autofluorescence levels and a FSC vs SSC profile comparable to those of AMØs from control mice, supporting that monocyte differentiation into pre-AMØs determined their translocation into the alveolar space. To confirm this hypothesis, intra-alveolarly-located cells were detected by i.t. administration of anti-CD45 antibodies, as described in **Figure 5B**. pre-AMØs and new-AMØs, but neither monocytes nor the majority of moCs, were stained with anti-CD45 (**Figure 5C**), confirming the intra-alveolar location of pre-AMØs. These results further support that new-AMØs were generated from monocytes, through an intermediate pre-AMØ population. pre-AMØs translocation into the alveolar space was therefore concomitant with the acquisition of autofluorescence and upregulation of Siglec-F and the M2 macrophage marker CD206 (**Figure 5D**).

### HDM allergy led to a reduced alveolar-capillary gas exchange surface and altered surfactant composition and biophysical properties

**Figure 6:**
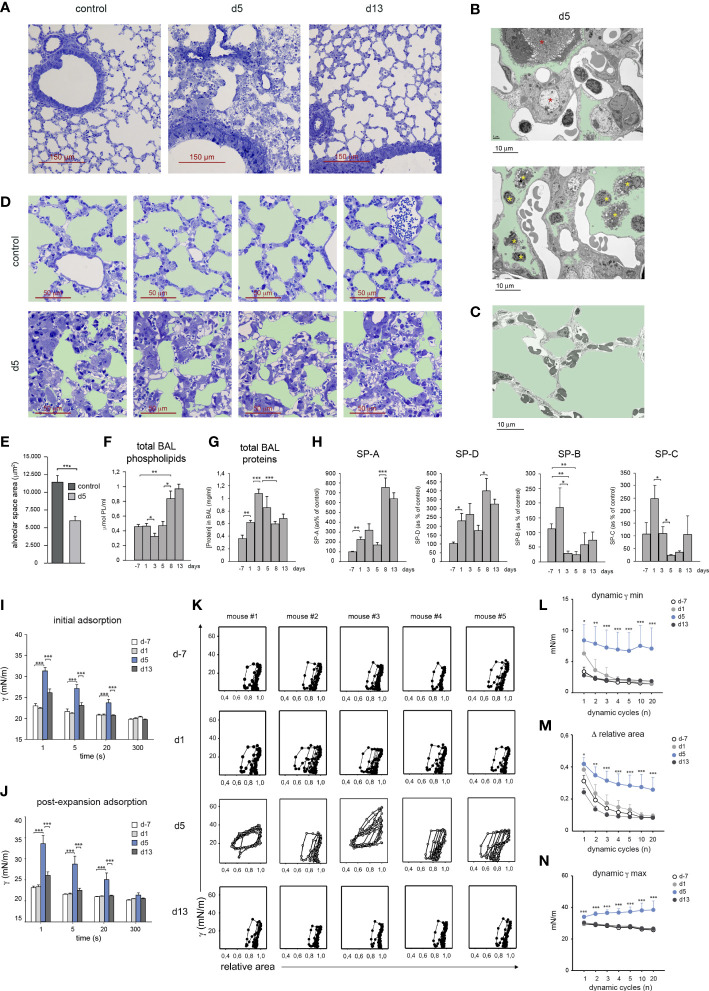
HDM allergy led to a reduced alveolar-capillary gas exchange surface and altered surfactant composition and biophysical properties. **(A)** Lung semi-thin sections at the indicated times; toluidine blue staining. **(B1, B2)** Ultra-thin lung sections analyzed by electron microscopy at d5 of HDM allergy, showing hypertrophic pneumocytes (**B1**, red asterisks) and leukocytes in the alveolar space (B2, yellow asterisks); light green: alveolar space. **(C)** Ultra-thin lung of a control non-allergic lung; light green: alveolar space. **(D)** Lung semi-thin sections from untreated mice and at d5; light green: alveolar space. **(E)** Quantification of the alveolar space areas defined in the micrographs shown in **(B)** Data expressed as mean alveolar space area/micrograph ± SD of 4 micrographs/condition. **(F, G)** Total BAL phospholipid **(F)** and protein **(G)** concentration in cell-free BAL supernatants at indicated times. **(H)** Analysis of surfactant proteins by Western blot and protein band quantification by densitometry, at indicated times. **(I, J)** Surface tension (γ) after initial interfacial adsorption **(I)**, and after bubble expansion and surfactant reextension **(J)**, upon injection of surfactant at the surface of a bubble air-liquid interface, measured by CBS in surfactant samples at indicated times. Surface tension at the interface measured immediately before and after surfactant injection was 70 and 30 mN/m. **(K)** Dynamic γ-area isotherms obtained during compression-expansion cycles measured by CBS. Changes in surface tension with respect to bubble relative area are shown for the 1^st^, 2^nd^, 3^rd^, 4^th^, 5^th^, 10^th^ and 20^th^ cycles. **(L)** Average minimum surface tension (γ min), **(M)** mean area reduction required to produce minimum surface tension (Δ relative area) and **(N)** average maximum surface tension (γ max), obtained during the dynamic isotherms described in **(I)**. In D-L, data are expressed as mean ± SEM of five mice/condition. Similar results were obtained in 3 independent experiments.

At d5, HDM allergy caused a severe bronchiolar inflammation characterized by increased mucus production and fibrosis (**Figure 1D**), as well as profound alterations in alveolar organization (**Figure 6A**), that included AMØ disappearance (**Figure 1B**) and massive alveolar eosinophilia (**Figure 1H**). In addition, electron microscopy studies revealed an extensive pneumocyte hypertrophy and leukocyte extravasation into the alveolar space at d5 (**Figures 6B1, B2**), not detectable in control lung (**Figure 6C**). Pneumocyte hypertrophy led to a marked thickening of alveolar wall and consequently to a significant reduction of the alveolar space and gas-exchange surface area (**Figures 6D, E**). By d13, the alveolar organization was essentially reestablished and bronchiolar inflammation was resolved (**Figure 6A**).

Since lung surfactant has an essential role in stabilizing the alveolar gas-aqueous interface and preventing lung collapse, we asked whether the disrupted alveolar organization and pneumocyte hypertrophy observed at the peak of HDM allergy, altered the composition and biophysical properties of lung surfactant. Total phospholipid concentration present in BAL remained unchanged until d5, but underwent a marked increase during the resolution of allergy (**Figure 6F**). In contrast, total BAL protein concentration increased during the induction of allergy and dropped during the resolution phase (**Figure 6G**). Surfactant-associated proteins SP-A and SP-D increased during the induction of the allergic reaction and more markedly from d8, i.e. during the resolution of HDM allergy, as assessed by western blot (**Figure 6H**). In contrast, SP-B and SP-C underwent a strong reduction by d5 reaching values significantly lower than those found in non-allergic mice (**Figure 6H**).

Based on these results, whether these changes in surfactant proteins affected the biophysical properties of lung surfactant was analyzed by captive bubble surfactometry (CBS), a method described in **Supplementary Figure 1**, allowing to assess the ability of a surfactant sample to form a surface-active film competent to produce and sustain a very low surface tension at the air-liquid interface of an air microbubble, mimicking an alveolar chamber. By monitoring surface tension changes, we analyzed the “initial adsorption” of the surfactant to the air-liquid interface, the ability of adsorbed surfactant to spread and re-equilibrate into an expanding interface, or “post-expansion adsorption”, and the ability of the formed film to produce the very low surface tensions (<5 mN/m) required to stabilize the lungs during compression-expansion cycles, or “dynamic isotherms”, that mimic breathing dynamics (19).

Surfactant from d-7 and d1 adsorbed (initial adsorption) and spread (post-expansion adsorption) at the air-liquid interface almost instantaneously, reducing surface tension from 〜70 mN/m to below 25 mN/m in less than 1 s (**Figures 6I, J**). In contrast, surfactant from d5 required more than 20 s to reach similar low surface tension values. Interestingly, fast interfacial adsorption, both initially and upon expansion, was restored in surfactant from d13. Upon compression-expansion cycles, interfacial films formed by surfactant from d-7 and d1 produced extremely low surface tensions at the end of every cycle (**Figures 6K, L**), with less than 20% area reduction from the second cycle (**Figures 6K, M**). These very low tension values were repeatedly achieved along 20 cycles, with very little hysteresis (assessed by the area enclosed between compression and expansion moieties of isotherms), indicating that the interfacial film had a high stability. Films formed by surfactant from d5 produced a deficient stabilization of the interface during compression-expansion cycles (**Figure 6K**). Only in some cases d5 films reached low enough surface tensions, yet requiring larger area reductions during a number of cycles, and showed considerably more hysteresis than films formed by surfactant from d-7 or d1. The low stability of films formed by surfactant obtained at the peak of HDM allergy also translated into the considerably higher maximum tension reached during the expansion phases (**Figures 6K, N**). Surfactant from d13 had fully restored dynamic isotherms and functional parameters, since in all cases films reached very low tensions (**Figure 6L**), with less than 20% area reduction (**Figure 6M**), while maintaining maximal tensions at the end of expansion (**Figure 6N**), that did not substantially differ from the equilibrium values produced at the end of the adsorption stage, along the 20 compression-expansion cycles applied.

These results revealed that surfactant obtained from mice during the peak of HDM allergy displayed a significant level of inactivation, characterized by slower interfacial adsorption and a reduced efficiency to attain and sustain very low surface tensions, raising the risk of alveolar collapse. Interestingly, during the resolution of the allergic reaction, the biophysical properties of surfactant were fully restored, as demonstrated by their high efficiency to form surface-active films maintaining very low surface tensions during breathing-like compression-expansion dynamics.

## Discussion

AMØs belong to the family of tissue-resident macrophages (resMØs), that share the expression of core lineage related genes determined during embryonic life, but acquire tissue-specific features dictated by tissue specific microenvironmental signals (16, 20).

resMØs present at birth are of embryonic origin, since they derive from yolk sac macrophages and fetal liver monocytes, these two precursor populations contributing differentially to resMØ subsets depending on their location (21). Embryonic resMØs self-maintain in the adult mice, but appear to be gradually, yet partially, replaced by adult monocyte-derived resMØs in the steady state, except for microglial, Langerhans and Kupffer cells (16, 22). In addition, recent experimental evidence supports that during infection, inflammation, or tissue damage, new resMØs, including AMØs, can be generated from adult BM monocytes (20, 22).

Our results demonstrate that HDM-induced allergy caused a massive disappearance of original embryonic AMØs, that were replaced a new population of monocyte-derived AMØs through an intermediate, intra-alveolar, pre-AMØ stage, as demonstrated by monocyte transfer experiments, assessment of intra-alveolarly-located cells, and analysis of AMØ regeneration in *Cx3cr1* ^cre^:*R26-yfp* chimeric mice. The monocyte to AMØ transition, involved a pre-AMØ stage, and was paralleled by the translocation into the alveolar space, acquisition of high levels of autofluorescence, upregulation of Siglec-F and CD206 and inactivation of CX3CR1 promoter, leading to CX3CR1 downregulation, as summarized in **Supplementary Figure 2**. We speculated that a fraction of monocytes recruited to a non-alveolar compartment, such as the iBALT, maintained CX3CR1 promoter activity and YFP locus rearrangement during their differentiation into lung mature moCs, that would participate in lung allergic inflammation, as reported (17). In contrast, new-AMØs would derive from monocytes recruited to an alveolar microenvironment, that would induce shut off monocyte CX3CR1 promoter, and drive monocyte differentiation into AMØs.

To our knowledge, AMØ disappearance, and long-term replacement of original AMØs by monocyte-derived AMØs, during airway allergy, described in the present study, were not previously reported. Interestingly replacement of embryonic AMØs by adult BM-derived AMØs has been reported during lung viral infections (23–26) or bleomycin-induced lung fibrosis (25), although data on the origin, degree of replacement and persistence of the newly-formed AMØ population remain controversial. During influenza virus infection, new-AMØs were proposed to derive either from non-monocytic BM precursors (23) or from monocytes (25). New-AMØs were proposed, but not demonstrated, to be monocyte-derived, during herpes virus infection (26) or experimental fibrosis (25). Besides, our results demonstrate that new monocyte-derived AMØs persisted at least three months after the induction of allergy. In contrast, during influenza virus infection, newly-formed monocyte-derived AMØs were claimed to be replaced in the long term by self-renewal the original embryonic population (23). After influenza virus infection or bleomycin-induced fibrosis, reported to cause a partial destruction of AMØs, new-AMØs coexisted in the long term with the remaining original AMØs (25). Interestingly, monocyte-derived AMØs, induced by influenza A infection, were recently demonstrated to persist at least 2 months after infection, and confer prolonged protection from *Streptococcus pneumoniae* infection due to increased production of IL-6 (27). In line with this study, recent experimental evidence has confirmed the concept that new AMØs formed during lung infectious or inflammatory processes develop from Ly6C^high^ monocytes recruited in a CCR2-dependent manner (28).

HDM allergy caused a profound alveolar disorganization involving AMØ death, pneumocyte hypertrophy and alterations in surfactant protein composition and biophysical properties. Dysfunction of alveolar system caused by allergic reactions was not previously reported. Nevertheless, AT2 cell hypertrophy was described in transgenic mice overexpressing IL-13 (29), a key mediator of lung Th2 immune diseases, such as asthma and fibrosis, and in a murine model of pulmonary fibrosis caused by TGF-β1 overexpression (30).

The hydrophilic proteins SP-A and SP-D are involved in lung innate immunity and regulation of surfactant homeostasis (31). SP-A and SP-D enhanced IL-4/IL-13-dependent M2 macrophage activation, promoting lung repair during helminth infection (32, 33). Therefore, the increased SP-A and SP-D levels at d8-d13 might contribute to lung tissue repair and alveolar reorganization during the resolution of HDM allergy. SP-B and SP-C hydrophobic surfactant proteins have a key role in the formation of the surface-active films at the air-liquid interface, required to reduce alveolar surface tension (3). Therefore, their low concentration at the peak of HDM allergy most likely contributed to the reduced efficiency of d5 surfactant to form surface-active films capable to maintain very low surface tensions, increasing the danger of alveolar collapse. Surfactant dysfunction was reported in murine models of lung infection (34, 35), and in ARDS (36) and asthmatic patients (37). Reduced amounts of SP-B and SP-C were described during airway allergy induced by *Aspergillus fumigatus* (38), in animal models of fibrosis (30), and in idiopathic fibrosis patients (39). A significant reduction in SP-B was associated with surfactant dysfunction and deficient lung mechanics in a mouse model of fibrosis (30), and SP-B deficiency was reported to impair surfactant function, resulting in respiratory failure (40). These data further support that surfactant dysfunction caused by HDM allergy was linked to the reduction in the surfactant proteins SP-B and SP-C.

Recruited monocyte-derived AMØs formed during inflammatory conditions have been claimed to retain the plasticity of monocytes, allowing them to adapt to the inflamed lung environment (28). Indeed, monocyte-derived AMØs provided a more efficient protection against infection, but can be detrimental by releasing profibrotic growth factors and chemokines, that cause the development of chronic lung diseases, such as fibrosis and chronic obstructive pulmonary disease (COPD) (28). Alveolar organization and surfactant function were restored after the resolution of HDM allergy, reflecting that newly-formed monocyte-derived AMØs are fully competent in maintaining surfactant recycling and function, and consequently alveolar homeostasis. However, whether these monocyte-derived AMØs contribute to the development of the allergic reaction and/or to the airway remodeling during the resolution of HDM-allergy remains to be assessed.

On the other hand, since AMØ function and survival require a continuous and finely regulated physical and biochemical interaction with pneumocytes (41), AMØ death caused by HDM allergy might result from disrupted alveolar organization and surfactant disfunction. In this regard, it can be speculated that pneumocyte hypertrophy caused by HDM-allergy might alter the expression of cell surface receptors mediating pneumocyte-AMØ interactions, such as CD200 and CD47, and compromise Gap junction-mediated bi-directional metabolic communication between pneumocytes and AMØs (42), that could ultimately lead to AMØ detachment from alveolar epithelial cells and AMØ death. On the other hand, in a mouse model of allergic asthma, HDM was reported to cause airway epithelial cell necroptosis, a process proposed to be mediated by the induction of death receptor ligands such as TNF and TRAIL (43). Moreover, intratracheal instillation of fine particles containing aluminum salts, causing chronic pulmonary inflammation, induced AMØ cell death by RIPK3-dependent necroptosis (44). Based on these reports, it can be speculated that AMØ death could also result from the induction of necroptosis during HDM-induced allergy. The mechanism by which AMØ die during airway allergy remains nevertheless to be defined.

In conclusion, our data support that the pathological disorders associated with allergic asthma not only result from bronchiolar inflammation, but additionally from a severe alveolar damage compromising surfactant function and an efficient gas exchange. These findings strongly suggest that new therapeutic strategies against asthma should be designed based on a combined treatment of bronchiolar inflammation and alveolar dysfunction.

## Data availability statement

The original contributions presented in the study are included in the article/**Supplementary Material**. Further inquiries can be directed to the corresponding authors.

## Ethics statement

The animal study was reviewed and approved by CNB Animal Care Committee (protocol 312/14).

## Author contributions

CaA designed the research, analyzed the data and wrote the manuscript. ML-B designed the research, performed experiments, analyzed the data and helped in preparing the manuscript. LF-L performed the majority of the experiments and analyzed the data. CG, ME, NA-L, LV, AV-P, LG-C, JD-A, GC and AH performed experiments. CC, BG-F and CM-F performed BAL biochemical analyses. JP-G and ChA analyzed the biophysical properties of lung surfactant. All authors contributed to the article and approved the submitted version.
