# Supplementary material for: Airway allergy causes alveolar macrophage death, profound alveolar disorganization and surfactant dysfunction
Source: Front Immunol. 2023 May 10;14:1125984. doi: 10.3389/fimmu.2023.1125984 (PMC10206250; doi:10.3389/fimmu.2023.1125984)
Supplement: Supplementary file 1 [file DataSheet_1.pdf]

Supplementary Material

Airway allergy causes alveolar macrophage death, profound alveolar disorganization and surfactant dysfunction

Lidia Feo-Lucas[1], Cristina Godio[1], María Minguito de la Escalera[1], Natalia Alvarez-Ladrón[1], Laura H. Villarrubia[1], Adrián Vega-Pérez[1,5], Leticia González-Cintado[1,6], Jorge Domínguez-Andrés[1,7], Belén García-Fojeda[2], Carlos Montero-Fernández[2], Cristina Casals[2], Chiara Autilio[2,3,8], Jesús Pérez-Gil[2,3], Georgiana Crainiciuc[4], Andrés Hidalgo[4], María López-Bravo[1,9]* and Carlos Ardavín[1*]

***Correspondence**: Carlos Ardavín (ardavin@cnb.csic.es) and María López-Bravo (mlbravo@cnb.csic.es).

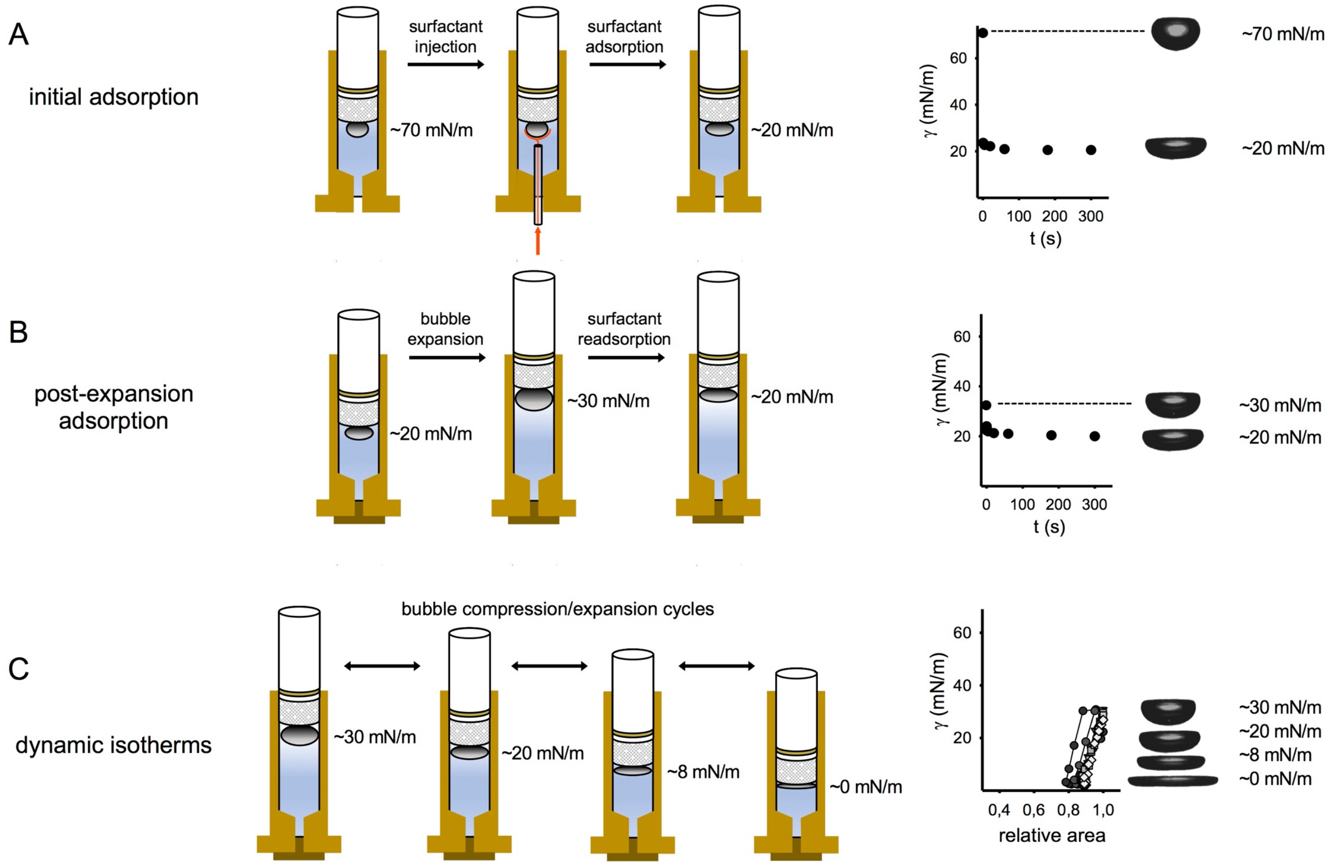

Supplementary Figure 1. Description of CBS experiments.

CBS allows testing surfactant activity during compression-expansion cycles that mimick the cyclic changes in alveolar volume during breathing. The ability of a surfactant sample to adsorb, re-expand and produce very low surface tension (γ) during compression, at 37°C, is evaluated at the air-liquid interface of an air microbubble, mimicking an alveolus, inside a chamber filled with a Tris buffer containing 150mM NaCl and 10% sucrose, to allow surfactant floating. The microbubble is compressed and expanded through changes in the hydrostatic pressure inside the chamber. Changes in the shape and size of the microbubble, caused by compression and expansion, are continuously recorded, permitting the analysis of changes in volume, area, and surface tension all along the experiment. (A) Initial adsorption is determined, after applying the surfactant sample onto the surface of the microbubble, by assessing the adsorption of the surfactant at the air-liquid interface, for 5 min. Control surfactant samples typically cause a surface tension reduction from ~ 70 mN/m (water surface tension) to ~ 23 mN/m in less than 1 s. (B) Post-expansion adsorption was determined after sealing the chamber, expanding the microbubble to a volume of 0.15 cm^3 (leading to an increase in surface tension to ~ 30 mN/m), and assessing the post-expansion readsorption of the surfactant for 5 min. Control surfactant samples typically cause the reduction of surface tension to the equilibrium value (~ 23 mN/m) in less than 1 s. (C) Dynamic isotherms were determined by applying a dynamic cycling by which the microbubble is subjected to 30 compression-expansion cycles in a minute. Changes in area and surface tension are measured over time and data represented as surface tension *versus* area isotherms.

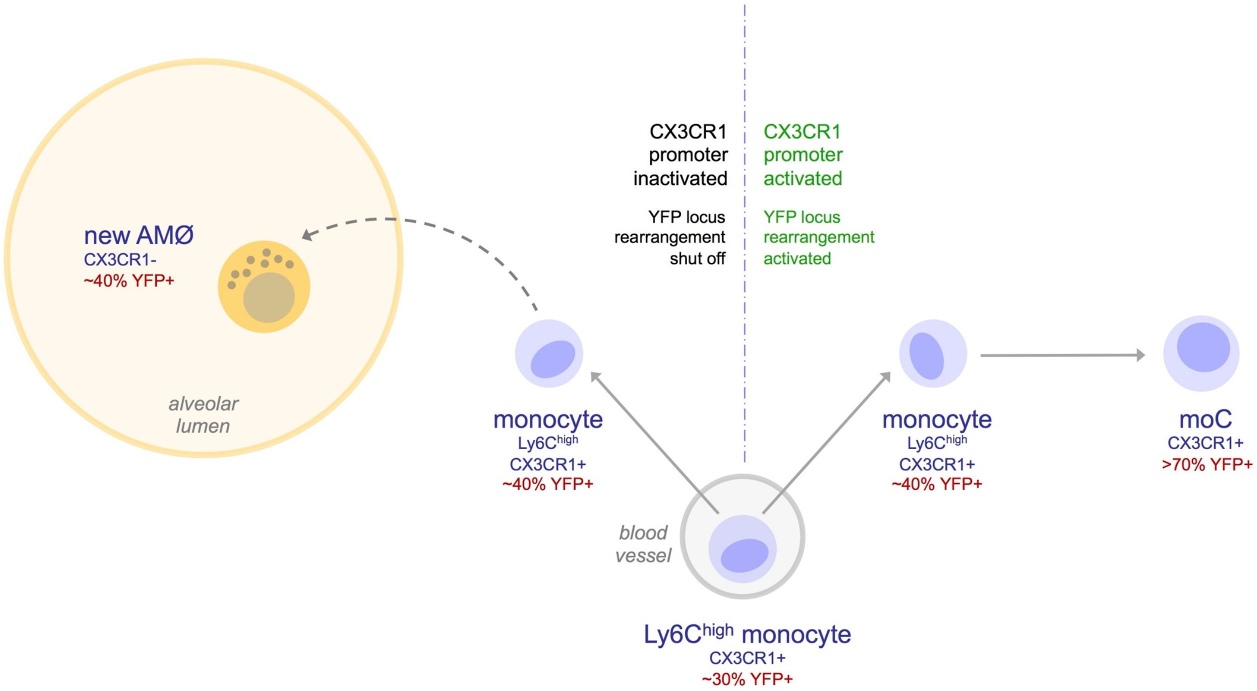

Supplementary Figure 2. Proposed model for the generation of new AMØs from Ly6C^{high} monocytes during HDM allergy, based on monocyte transfer experiments and analysis of *Cx3cr1*^{cre}:*R26-yfp*/CD45.1 chimeras.
Transition from Ly6C^{high} monocytes to new AMØs would be concomitant with the inactivation of the CX3CR1 promoter and therefore to the shut off of Cre-mediated rearrangement of the YFP locus. During HDM allergy a fraction of mo-Cs would maintain the CX3CR1 promoter active and efficiently rearrange the YFP locus, due to their extended life span, and to the location where they were recruited. In contrast, in mo-Cs giving rise to new AMØs, the CX3CR1 promoter would be shut off, and the new AMØ population would display a similar YFP positivity than the Ly6C^{high} monocytes from which they derive.
